# Combined Use of Phenotypic Screening and of a Novel Commercial Assay (REALQUALITY Carba-Screen) for the Rapid Molecular Detection of Carbapenemases: A Single-Center Experience

**DOI:** 10.3390/diagnostics14151599

**Published:** 2024-07-25

**Authors:** Federica Novazzi, Gabriele Arcari, Francesca Drago Ferrante, Sara Boutahar, Angelo Paolo Genoni, Davide Carcione, Gianluca Cassani, Paolo Gigante, Mattia Carbotti, Riccardo Capuano, Renée Pasciuta, Nicasio Mancini

**Affiliations:** 1 Laboratory of Medical Microbiology and Virology, University Hospital of Varese, 21100 Varese, Italy; federica.novazzi@uninsubria.it (F.N.); gabriele.arcari@uninsubria.it (G.A.);; 2 Department of Medicine and Technological Innovation, University of Insubria, 21100 Varese, Italy; 3 Laboratory of Clinical Microbiology and Virology, ASST Valle Olona, 21013 Gallarate, Italy

**Keywords:** molecular detection, surveillance programs, antimicrobial resistance, phenotypic screening, carbapenemases

## Abstract

Carbapenem resistance is a serious public health threat, causing numerous deaths annually primarily due to healthcare-associated infections. To face this menace, surveillance programs in high-risk patients are becoming a widespread practice. Here we report the performance of the combined use of a recently approved commercial multiplex real-time PCR assay (REALQUALITY Carba-Screen kit) with conventional phenotypic screening. In this three-month study, 479 rectal swabs from 309 patients across high-risk units were evaluated by combining the two approaches. Although the molecular assay showed a higher positivity rate than phenotypic screening (7.1% vs. 5%), it should be noted that the molecular method alone would have missed eight carbapenem-resistant isolates, while using only phenotypic screening would not have detected sixteen isolates. This demonstrates the complementary strengths of each method. Our study confirms the need for a combined approach to maximize the possible clinical impact of this kind of screening, ensuring a more comprehensive detection of resistant strains.

## 1. Introduction

Antimicrobial resistance is a major global threat to public health, affecting humans, animals, and the environment [1]. The 2023 European Centre for Disease Prevention and Control (ECDC) antimicrobial resistance surveillance report, referring to data from strains isolated in 2021, describes a heterogeneous antimicrobial resistance condition [2]. However, while the spread of resistance varies according to bacterial species, antimicrobial group, and geographical region, carbapenems are typically used as the hallmark for the spread of antimicrobial resistance. Theoretically, all microorganisms have the potential to reach carbapenem resistance. However, considering clinical relevance, the most meaningful carbapenem-resistant microorganisms belong to three orders: *Enterobacterales* (e.g., *Klebsiella pneumoniae* and *Escherichia coli*), *Pseudomonadales* (e.g., *Pseudomonas aeruginosa*), and *Moraxellales* (e.g., *Acinetobacter baumannii*).

The European epidemiology is characterized by a significant carbapenem resistance rate, with prevalences which can be over 50% for *K. pneumoniae*, *P. aereuginosa*, and *A. baumannii* in some countries [2]. Resistance rates are exceptionally high in Italy, where the increasing prevalence of carbapenemase-producing Gram-negative bacteria causes many deaths each year, mostly due to healthcare-associated infections [3].

Carbapenemases are broad-spectrum beta-lactamases which can hydrolyze carbapenem antibiotics characterized by adaptable hydrolytic capacities which allow them to overcome inhibition by beta-lactam-based beta-lactamases inhibitors (i.e., clavulanic acid, sulbactam, and tazobactam) [4] and have a leading role in the epidemiology of carbapenem-resistant microorganisms.

According to their chemical structure, carbapenemases can be assigned to Ambler class A (such as KPC), B (metallo-beta-lactamases such as NDM, VIM, and IMP), and D (further distinguished into Group I carbapenemases [OXA-23-like, OXA-24-like, OXA-51-like, and OXA-58-like] commonly identified in *A. baumannii* [5] and Group II carbapenemases [OXA-48-like] commonly identified in *Enterobacterales* [6]).

Being a major threat in hospital settings, routine surveillance programs (e.g., rectal swabs at admission and once a week) are a tool to prevent the spread of resistant microorganisms, especially in high-risk categories of patients [7,8].

Several methods featuring different sensitivity and economic cost values, typically based either on phenotypic or genotypic approaches, are available for routine screening of carbapenem-resistant Gram-negative bacteria carriage.

Phenotypic approaches include the direct inoculation onto MacConkey agar with the addition of carbapenem discs [9] or the use of selective chromogenic media [10]. Carbapenemase production can be inferred through carbapenemase inhibition phenotypic tests (i.e., mCIM and eCIM test [11,12]) or through commercially available lateral flow immunochromatographic assays for the detection of the most common carbapenemases, including OXA-48, KPC, NDM, VIM, and IMP [13]. Conversely, several molecular tests based on the identification of resistance genes (either from culture or directly from biological samples) are commercially available [14]. These assays differ in DNA extraction protocols, levels of process automation, and turnaround times (usually ranging from 1 to 3 h) [15,16,17,18,19].

Colistin (polymyxin E), on the other hand, is a polycationic antimicrobial peptide which serves as a “last-resort antibiotic” for the treatment of multi-drug-resistant infections caused by multi-drug-resistant (MDR) Gram-negative bacteria which fell into disuse owing to the rapid and frequent onset of colistin-induced renal damage [20,21]. The current increase in infections caused by MDR Gram-negative bacteria (above all carbapenem-resistant *K. pneumoniae*, *P. aeruginosa*, and *A. baumannii*) prompted the reintroduction of colistin in clinical practice, yielding to an upsurge in colistin resistance [20]. The most frequent mechanisms underlying colistin resistance in carbapenem-resistant *K. pneumoniae*, *P. aeruginosa*, and *A. baumannii* are linked to chromosomal mutation resulting in an augmented expression of lipopolysaccharide-modifying genes (i.e., the *phoP/phoQ* two component system) [20]. However, plasmid-mediated colistin resistance conferred by the *mcr* genes is an increasing threat [22].

In this study, we report the performance of the combined use of a recently licensed commercial multiplex quantitative real-time PCR assay (REALQUALITY Carba-Screen kit) with conventional phenotypic screening. Specifically, we aim to underscore the novelty of integrating phenotypic screening with molecular assays. This combined approach enhances the rapid molecular detection of carbapenemases, improves diagnostic accuracy, and impacts clinical decision-making. By leveraging the strengths of both phenotypic and molecular methods, our study presents a novel strategy that could set a new standard for the rapid and accurate diagnosis of carbapenemase-producing organisms in clinical settings.

## 2. Materials and Methods

### 2.1. Patients and Samples

The study was conducted over a three-month period from August 2023 to October 2023 at the University Hospital of Varese, in North-West Italy close to the Swiss border, and included 479 rectal swabs from 309 patients admitted to several high-risk units (intensive care units, infectious disease unit, neonatal pathology, pediatrics, hematology, general surgery, cardiac surgery, neurosurgery). The patients were sampled at admission and every 5–7 days during hospitalization.

All rectal swabs that arrived in the three-month period at our laboratory for multi-drug-resistant screening were included in this study. However, molecular screening tests were carried out on leftovers samples; hence, this testing did not influence clinical practice.

### 2.2. Phenotypic Screening of Carbapenem Resistance

All swabs were directly inoculated onto MacConkey agar (bioMérieux, Marcy l’Étoile, France) plates supplemented with an MEM 10 μg disc, and the plates were incubated for up to 48 h at 35 °C [23]. The results were interpreted according to the current European Committee on Antimicrobial Susceptibility Tests (EUCAST) breakpoint guidelines [24]. Testing for carbapenemases was warranted whenever the zone diameter of MEM was <25 mm in all potential carbapenemase producers including *K. pneumoniae*, *A. baumannii*, *P. aeruginosa*, and *E. coli*. All resistant isolates were identified at the species level through MALDI-TOF using VITEK MS (bioMerieux, Marcy l’Étoile, France).

### 2.3. Antimicrobial Susceptibility Testing

All carbapenem-resistant isolates were tested by the VITEK^®^2 compact system (bioMérieux, Marcy l’Étoile, France). To perform the test, a homogeneous suspension of microorganisms at 0.5 McFarland standard density was prepared from pure culture. The VITEK^®^2 AST-N397 card was used which includes amikacin, amoxicillin–clavulanic acid, cefotaxime, cefepime, ceftazidime, ciprofloxacin, gentamicin, ertapenem, imipenem, meropenem, piperacillin–tazobactam, trimethoprim–sulfamethoxazole, ceftazidime–avibactam, and ceftolozane–tazobactam. The MIC values were interpreted according to EUCAST clinical breakpoints (v 13.0) [24].

### 2.4. Molecular Test: REALQUALITY Carba-Screen Kit AB ANALITICA Multiplex Quantitative Real-Time PCR

DNA was extracted directly from all rectal swabs with the Microlab STARlet IVD platform (Hamilton Company, Reno, NV, USA) using the STARMag 96 × 4 Universal Cartridge Kit (Seegene, Seoul, Republic of Korea) according to the manufacturer’s instructions. The extracted DNA was amplified by REALQUALITY Carba-Screen kit (AB ANALITICA, Padua, Italy) using the QuantStudio 5™ qPCR, 96 well, 0.2 mL instrument (Applied Biosystems, Waltham, MA, USA) according to the manufacturer’s instructions.

The kit includes a first screening step (Mix Carba-Screen) to identify samples positive for class A, B, and D carbapenemases, including *Acinetobacter* spp.-related OXA genes (*bla*_OXA-23_-like, *bla*_OXA-24_-like, *bla*_OXA-51_-like with promoter IS*Aba1*, and *bla*_OXA-58_-like, henceforth defined as *Ac*OXA). The positive samples undergo a second step (Mix Carba B and Mix Carba A + D) to identify the specific resistance gene involved.

Molecular analyses were performed a posteriori on leftovers samples, hence did not influence clinical practice.

### 2.5. Statistical Analyses

Positivity rate was calculated by dividing the number of positive cases detected by phenotypic testing, molecular testing, or both approaches combined by the total number of samples tested and it was expressed as a percentage.

### 2.6. Ethical Aspects

This work was performed in agreement with the latest version of the Helsinki Declaration.

## 3. Results

**Figure 1:**
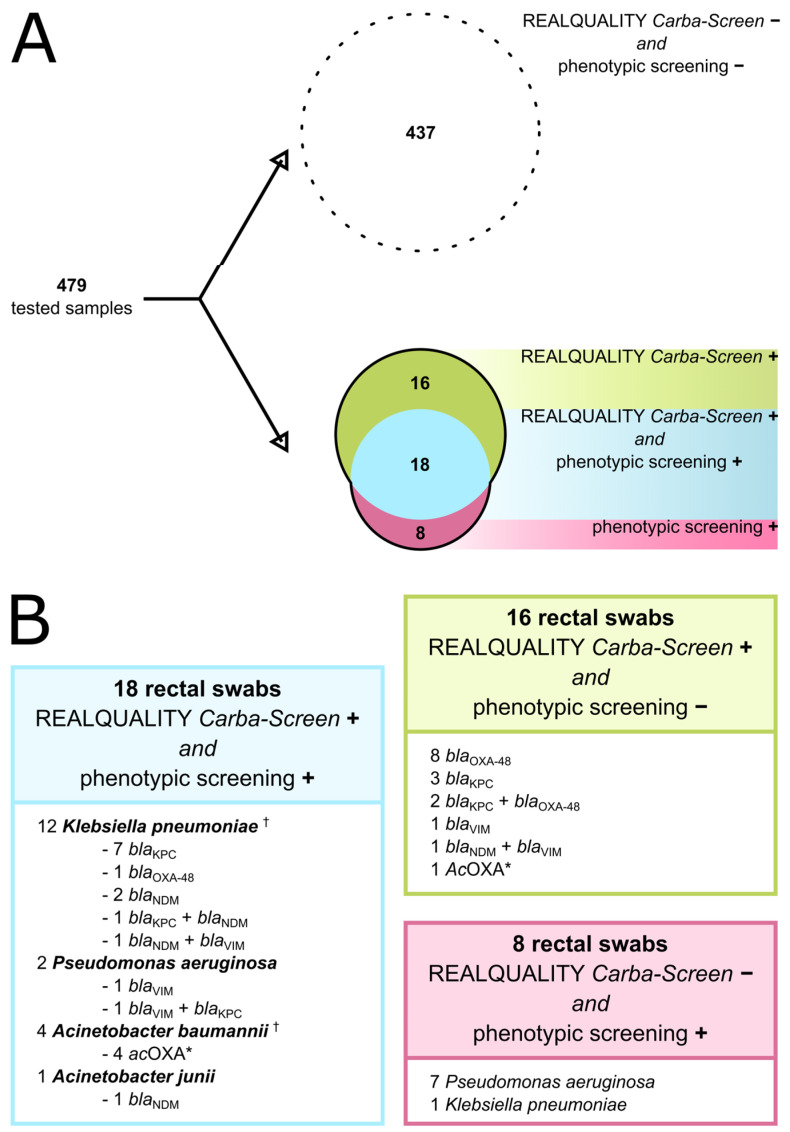
**Schematic representation of the results.** Panel (**A**) Distribution of tested samples categorized by assay outcomes. The dotted circle contains swabs negative to both phenotypic and molecular assays. Numbers within the green and pink sets of the Venn diagram represent swabs positive in the molecular and phenotypic assays, respectively. The cyan intersection displays swabs positive in both assays. Panel (**B**) Breakdown of positive samples by detection assay. †: two microorganisms identified from the same rectal swab; * *Ac*OXA: *bla*_OXA-23_-like, *bla*_OXA-24_-like, *bla*_OXA-51_-like, and *bla*_OXA-58_-like.

Among the 309 patients involved in the study, the median age was 65 years (IQR 0–91), and 119 (38.5%) were female. A total of 479 swabs were tested, of which 26 (5.1%) grew possible carbapenem-resistant isolates by disc diffusion screening, later confirmed by VITEK^®^ 2 testing (Figure 1A). The carbapenem-resistant isolates included twelve (44.4%) *K. pneumoniae*, nine (33.3%) *P. aeruginosa*, three (11.1%) *A. baumannii*, and one (3.7%) *Acinetobacter junii*. In a single case (3.7%), a multiple colonization by carbapenem-resistant *K. pneumoniae* and *A. baumannii* was detected (isolates 376k and 376a, Table 1 and Table 2).

The same swabs were processed in parallel using the REALQUALITY Carba-Screen AB ANALITICA qPCR kit, and carbapenemase genes were detected in 34 (6.7%) samples. More in detail, eighteen swabs (52.9%) were positive using both the culture-based and the molecular assay, with the detection of nine (50.0%) *bla*_KPC_, five (27.8%) *bla*_NDM_, four (22.2%) *Ac*OXA, three (16.7%) *bla*_VIM_, and one (5.5%) *bla*_OXA-48_ (Figure 1A,B, Table 1). In three rectal swabs, multiple resistance genes were co-detected: in one case *bla*_VIM_ + *bla*_KPC_, in one case *bla*_VIM_ + *bla*_NDM_, and in one case *bla*_KPC_ + *bla*_NDM_. Analyzing the swabs positive in both the molecular and phenotypic assays, *K. pneumoniae* was the most represented isolate, being identified in twelve rectal swabs with the detection of seven (58.3%) *bla*_KPC_, three (25.0%) *bla*_NDM_, one (8.3%) *bla*_OXA-48_, one (8.3%) *bla*_NDM_ in association with *bla*_VIM_, and one (8.3%) *bla*_KPC_ in association with *bla*_NDM_.

Sixteen additional swabs were positive only using the molecular assay, whereas no carbapenem-resistant isolates were detected in culture. More in detail, eight samples (50%) were positive for *bla*_OXA-48_, seven (38.9%) for *bla*_KPC_, two (11.1%) for *bla*_VIM_, two (11.1%) for *bla*_NDM_, and one for *Ac*OXA (6.2%, Figure 1, Table 1). Multiple genes were co-detected in three cases, including *bla*_KPC_ and *bla*_OXA-48_ in two (12.5%) swabs and *bla*_NDM_ and *bla*_VIM_ in one (6.25%).

Finally, eight swabs (30.8%) were positive for carbapenem-resistant isolates only in the culture-based assay, of which seven (87.5%) were *P. aeruginosa* and one (12.5%) was *K. pneumoniae* (Figure 1A,B, Table 1 and Table 2). In the present study, the REALQUALITY Carba-Screen AB ANALITICA qPCR featured a positivity rate of 7.1% (34/479) for the identification of carbapenemase genes in all sample swabs, while the culture-based screening method demonstrated a positivity rate of 5% (24/479). A combination of both the molecular assay to identify carbapenemase genes and of the culture-based screening method to identify carbapenem resistance would have yielded the highest positivity rate (8.7%).

Microbial colonization by MDR pathogens occurring 8 to 30 days after hospitalization was reported in 7/309 (2.3%) of the patients included in the surveillance. In this context, *P. aeruginosa* was the most significant pathogen as it was identified as the causative agent of 5/7 (71.4%) of the hospital colonization cases, whereas *A. baumannii* (carrying an *Ac*OXA) and *A. junii* (carrying *bla*_NDM_) were the other identified pathogens. Only one patient was already colonized with MDR *P. aeruginosa* upon ICU admission. This highlights the importance of timely and accurate detection of MDR colonization for effective infection control and patient management in high-risk settings.

## 4. Discussion

Carbapenems play a crucial role against MDR Gram-negative pathogens. While there is no univocal consensus about the epidemiological impact of different mechanisms of carbapenem resistance [25,26,27], rectal carriage of carbapenem-resistant Gram-negative microorganisms is an important risk factor for developing carbapenem-resistant Gram-negative infections [28], which pose a greater risk of death for the patient [29]. For this reason, the present spread of MDR pathogens, especially in hospital settings, makes compelling the prompt and early identification of resistance especially in at-risk categories of patients.

In this study, we evaluated the performance of the combined use of a novel commercially available molecular-based assay (REALQUALITY Carba-Screen kit) for the screening of carbapenemase genes and of a conventional phenotypic assay. As of today, phenotypic testing strategies to identify carbapenem-resistant isolates are still more cost-effective and widely used [30]. However, these may suffer from several drawbacks which may lead to the failure in detecting carbapenem-resistant microorganisms, such as for example when their load is very low. To address this issue, incorporating a pre-enrichment step for rectal swabs could be beneficial. However, a step forward could be made by integrating the culture-based screening with a molecular-based assay, such as the one used in this study. Some studies have already addressed this point in the literature, evidencing the advantage offered by the combined approach [11,31]. More in detail, the molecular assay used in this study is characterized by two potential further advantages, namely the detection of *mcr* and of the *Ac*OXA genes, a feature laying the groundwork for future comparative analyses among different molecular assays which were not carried out in the present study.

Coherently with studies analyzing the performances of molecular-based antimicrobial-resistance screening platforms, the REALQUALITY Carba-Screen was able to identify a higher number of potential carbapenemase-producing isolates than the phenotypic culture-based screening (Figure 1A,B) [32]. This characteristic is of note in *bla*_OXA-48_-positive swabs (six identified only by REALQUALITY Carba-Screen), plausibly owing to the minor carbapenem hydrolytic activity of the encoded enzyme [33]. Moreover, the REALQUALITY Carba-Screen was able to identify *bla*_KPC_ in five swabs testing negative for phenotypic screening. This inconsistency could be a consequence of a low *bla*_KPC_ gene load in the swab, leading to an inexact phenotypic screening, or to the presence of one of the ceftazidime–avibactam-resistant meropenem-susceptible KPC variants [34]. Under this perspective, several approaches were proposed to improve performance in the carbapenemase detection method by standard culture such as, for example, that at least 30 µL of flocked buffer (Liquid Amies Elution) is sown after vortexing [35].

Another potential feature of improvement in the phenotypic screening lies in the correct choice of the combination medium antibiotic to be used, such as MacConkey agar + two antimicrobial disks (a 10 µg meropenem disc and a 10 + 4 µg ceftazidime–avibactam disc) coupled with a chromogenic agar. This phenotypic approach could broaden the landscape of identifiable microorganisms, allowing to search for carbapenem-resistant microorganisms, for bacteria with reduced susceptibility to carbapenems, and for carbapenem-susceptible ceftazidime–avibactam-resistant KPC variants [36,37]. While this study was not conducted in a real-life setting, the implementation of the molecular assay in the screening algorithm could bring several advantages in a laboratory routine, such as a decrease in the turnaround time and a higher sensitivity [32]. These features make molecular screening for MDR pathogen colonization a strong support to MDR outbreak control.

However, while molecular screening tests show promise, they are still burdened by some notable limitations. For instance, relying on amplification rather than on whole-genome sequencing, they cannot detect every resistance determinant. This limit mainly arises in *P. aeruginosa*, since in this species, carbapenem resistance is typically a multi-factorial phenomenon [38]. In our evaluation, the molecular assay was able to identify only two carbapenem-resistant strains, as compared to nine identified by phenotypic screening (Figure 1B). Additionally, if not properly carried out, molecular assays can lead to ambiguous conclusions. For instance, during this evaluation, we identified two peculiar occurrences: one *A. junii* strain carrying the *bla*_NDM_ gene, and one *P. aeruginosa* carrying both *bla*_VIM_ and *bla*_KPC_, with the latter only anecdotally reported. These two events are emblematic of how phenotypic and molecular assays should be combined to produce solid and meaningful results.

Additionally, molecular tests cannot accurately determine the minimum inhibitory concentration (MIC) for antibiotics and, while their reliability in demonstrating effectiveness with certain antimicrobial molecules (i.e., carbapenems) is high, there remains significant room for improvement in other settings, such as in the case of novel beta-lactam–non-beta-lactam beta-lactamase inhibitor combinations.

## 5. Conclusions

This study, while highlighting that molecular assays are more sensitive and specific than culture for the detection of carbapenemase genes in clinical samples, confirms that as of today, phenotypic antimicrobial-susceptibility testing cannot be left out of consideration. Phenotypic methods remain crucial for providing comprehensive information on the antimicrobial resistance profile, which is essential for guiding appropriate therapeutic strategies.

Here, by highlighting the novelty of the integrative approach combing phenotypic screening with the REALQUALITY Carba-Screen assay, we aim to prompt a novel way to perform screening for MDR microorganisms. This combined approach enhances rapid molecular detection of carbapenemases, improves diagnostic accuracy, and impacts clinical decision-making. By leveraging the strengths of both phenotypic and molecular methods, our study presents a novel strategy that could set a new standard for the rapid and accurate diagnosis of carbapenemase-producing organisms in clinical settings.

In the near future, a combined use of molecular and phenotypic screening is foreseeable, and their combination needs to be assessed according to the specific clinical need addressed and available economic resources. Integrating these methods can enhance diagnostic accuracy, improve patient outcomes, and optimize infection control measures. Overall, the REALQUALITY Carba-Screen assay (CE-IVD marked test) represents a possible option to be integrated into clinical screening of the major carbapenemases. This integration can help in early detection and appropriate management of infections caused by carbapenem-resistant organisms, ultimately contributing to better healthcare outcomes.

## Figures and Tables

**Table 1:** **Detailed description of the microorganisms identified in this study.** Specification of data from analyzed samples testing positive in the molecular REALQUALITY Carba-Screen assay, in the phenotypic assay, or both. Bold **P**s in columns display the observation of an antimicrobial resistance gene in the sample. **ND**: Not Detected, * *Ac*OXA: *bla*_OXA-23_-like, *bla*_OXA-24_-like, *bla*_OXA-51_-like, and *bla*_OXA-58_-like.

Sample Id	REALQUALITY *Carba-Screen*	*Ac*OXA *	*mcr*	*bla*_KPC_	*bla*_OXA-48_	*bla*_NDM_	*bla*_IMP_	*bla*_VIM_	Phenotypic Screening	Microorganism
274	**Positive**	**P**	-	-	-	-	-	-	**Positive**	*A. baumannii*
376a	**Positive**	**P**	-	-	-	-	-	-	**Positive**	*A. baumannii*
416	**Positive**	**P**	-	-	-	-	-	-	**Positive**	*A. baumannii*
465	**Positive**	**P**	-	-	-	-	-	-	**Positive**	*A. baumannii*
484	**Positive**	**P**	-	-	-	-	-	-	Negative	ND
188	**Positive**	-	-	**P**	-	-	-	-	**Positive**	*K. pneumoniae*
200	**Positive**	-	-	**P**	-	-	-	-	**Positive**	*K. pneumoniae*
249	**Positive**	-	-	**P**	-	-	-	-	**Positive**	*K. pneumoniae*
398	**Positive**	-	-	**P**	-	-	-	-	**Positive**	*K. pneumoniae*
21	**Positive**	-	-	**P**	-	-	-	-	Negative	ND
102	**Positive**	-	-	**P**	-	-	-	-	Negative	ND
180	**Positive**	-	-	**P**	-	-	-	-	Negative	ND
432	**Positive**	-	-	**P**		-	-	-	**Positive**	*K. pneumoniae*
455	**Positive**	-	-	**P**		-	-	-	**Positive**	*K. pneumoniae*
358	**Positive**	-	-	**P**	-	-	-	-	Negative	ND
379	**Positive**	-	-	**P**	-	-	-	-	Negative	ND
64	**Positive**	-	-	**P**	-	-	-	-	**Positive**	*K. pneumoniae*
191	**Positive**	-	-	**P**	-	-	-	**P**	**Positive**	*P. aeruginosa*
376k	**Positive**	-	-	**P**	-	**P**	-	-	**Positive**	*K. pneumoniae*
434	**Positive**	-	-	**P**	**P**	-	-	-	Negative	ND
447	**Positive**	-	-	**P**	**P**	-	-	-	Negative	ND
452	**Positive**	-	-	-	**P**	-	-	-	Negative	ND
479	**Positive**	-	-	-	**P**	-	-	-	Negative	ND
489	**Positive**	-	-	-	**P**	-	-	-	Negative	ND
429	**Positive**	-	-	-	**P**	-	-	-	**Positive**	*K. pneumoniae*
291	**Positive**	-	-	-	**P**	-	-	-	Negative	ND
293	**Positive**	-	-	-	**P**	-	-	-	Negative	ND
294	**Positive**	-	-	-	**P**	-	-	-	Negative	ND
359	**Positive**	-	-	-	-	**P**	-	-	**Positive**	*A. junii*
30	**Positive**	-	-	-	-	**P**	-	-	**Positive**	*K. pneumoniae*
56	**Positive**	-	-	-	-	**P**	-	-	**Positive**	*K. pneumoniae*
3	**Positive**	-	-	-	-	**P**	-	**P**	**Positive**	*K. pneumoniae*
70	**Positive**	-	-	-	-	**P**	-	**P**	Negative	ND
6	**Positive**	-	-	-	-	-	-	**P**	**Positive**	*P. aeruginosa*
104	**Positive**	-	-	-	-	-	-	**P**	Negative	ND
25	Negative	-	-	-	-	-	-	-	**Positive**	*K. pneumoniae*
71	Negative	-	-	-	-	-	-	-	**Positive**	*P. aeruginosa*
115	Negative	-	-	-	-	-	-	-	**Positive**	*P. aeruginosa*
139	Negative	-	-	-	-	-	-	-	**Positive**	*P. aeruginosa*
320	Negative	-	-	-	-	-	-	-	**Positive**	*P. aeruginosa*
375	Negative	-	-	-	-	-	-	-	**Positive**	*P. aeruginosa*
466	Negative	-	-	-	-	-	-	-	**Positive**	*P. aeruginosa*
506	Negative	-	-	-	-	-	-	-	**Positive**	*P. aeruginosa*

**Table 2:** Antibiotic susceptibility and MIC to meropenem of the strains identified by phenotypic screening.

Sample Id	Microorganism	AK	CM	CIP	AMC	CAZ	CTX	FEP	MEM		IMI	P/T	CZA	C/T	SXT
274	*A. baumannii*	**R**	**R**	**R**	NA	NA	NA	NA	**R**	>8	**R**	NA	NA	NA	**R**
376a	*A. baumannii*	**R**	**R**	**R**	NA	NA	NA	NA	**R**	>8	**R**	NA	NA	NA	**R**
416	*A. baumannii*	NA	**R**	**R**	NA	NA	NA	NA	**R**	>8	**R**	NA	NA	NA	**R**
465	*A. baumannii*	**R**	**R**	**R**	NA	NA	NA	NA	**R**	>8	**R**	NA	NA	NA	**R**
359	*A. junii*	**R**	S	I	NA	NA	NA	NA	I	8	I	NA	NA	NA	S
3	*K. pneumoniae*	NA	S	**R**	**R**	**R**	**R**	**R**	**R**	>8	**R**	**R**	NA	NA	**R**
25	*K. pneumoniae*	S	S	**R**	**R**	**R**	**R**	**R**	**R**	>8	**R**	**R**	S	NA	**R**
30	*K. pneumoniae*	NA	S	**R**	**R**	**R**	**R**	**R**	**R**	>8	**R**	**R**	NA	NA	**R**
56	*K. pneumoniae*	NA	S	**R**	**R**	**R**	**R**	**R**	**R**	>8	**R**	**R**	**R**	**R**	**R**
64	*K. pneumoniae*	S	S	**R**	**R**	**R**	**R**	**R**	**R**	>8	**R**	**R**	S	**R**	S
188	*K. pneumoniae*	S	S	**R**	**R**	**R**	**R**	**R**	**R**	>8	**R**	**R**	NA	NA	**R**
200	*K. pneumoniae*	S	S	**R**	**R**	**R**	**R**	**R**	**R**	>8	**R**	**R**	NA	NA	**R**
249	*K. pneumoniae*	S	S	**R**	**R**	**R**	**R**	**R**	**R**	>8	**R**	**R**	NA	NA	**R**
376k	*K. pneumoniae*	NA	**R**	**R**	NA	**R**	**R**	**R**	**R**	>8	**R**	**R**	NA	NA	**R**
398	*K. pneumoniae*	NA	**R**	**R**	NA	**R**	**R**	**R**	**R**	>8	**R**	**R**	NA	NA	**R**
429	*K. pneumoniae*	S	S	S	**R**	NA	**R**	NA	**R**	>8	**R**	**R**	S	NA	S
432	*K. pneumoniae*	NA	S	**R**	**R**	**R**	**R**	**R**	**R**	>8	**R**	**R**	S	**R**	**R**
455	*K. pneumoniae*	S	S	**R**	**R**	**R**	**R**	**R**	**R**	>8	**R**	**R**	NA	NA	NA
6	*P. aeruginosa*	NA	NA	**R**	NA	**R**	NA	**R**	**R**	>8	**R**	**R**	NA	NA	NA
71	*P. aeruginosa*	S	NA	I	NA	**R**	NA	I	I	4	**R**	I	NA	NA	NA
115	*P. aeruginosa*	S	NA	I	NA	**R**	NA	**R**	I	8	**R**	**R**	**R**	**R**	NA
139	*P. aeruginosa*	S	NA	I	NA	**R**	NA	I	**R**	>8	**R**	**R**	NA	NA	NA
191	*P. aeruginosa*	NA	NA	**R**	NA	**R**	NA	**R**	**R**	>8	**R**	**R**	NA	NA	NA
320	*P. aeruginosa*	S	NA	**R**	NA	I	NA	I	I	8	**R**	NA	NA	NA	NA
375	*P. aeruginosa*	S	NA	**R**	NA	**R**	NA	**R**	I	8	**R**	I	**R**	S	NA
466	*P. aeruginosa*	S	NA	**R**	NA	I	I	**R**	**R**	>8	**R**	**R**	NA	NA	NA
506	*P. aeruginosa*	S	NA	I	NA	**R**	NA	**R**	**R**	>8	**R**	**R**	NA	NA	NA

**S:** Susceptible, standard dosing regimen, **I**: Susceptible, increased exposure, **R:** Resistant, **NA**: not assessed, **AK**: amikacin, **CM**: gentamycin, **CIP**: ciprofloxacin, **AMC**: amoxicillin–clavulanic acid, **CAZ**: ceftazidime, **CTX**: cefotaxime, **FEP**: cefepime, **MEM**: meropenem, **IMI**: imipenem, **P/T**: piperacillin–tazobactam, **CZA**: ceftazidime–avibactam, **C/T**: ceftolozane–tazobactam, **SXT**: trimethoprim–sulfamethoxazole.

## Data Availability

The raw data supporting the conclusions of this article will be made available by the authors on request.
